# Diagnosis, Treatment and Prevention of Sarcopenia in Hip Fractured Patients: Where We Are and Where We Are Going: A Systematic Review

**DOI:** 10.3390/jcm9092997

**Published:** 2020-09-17

**Authors:** Gianluca Testa, Andrea Vescio, Danilo Zuccalà, Vincenzo Petrantoni, Mirko Amico, Giorgio Ivan Russo, Giuseppe Sessa, Vito Pavone

**Affiliations:** 1 Department of General Surgery and Medical Surgical Specialties, Section of Orthopaedics and Traumatology, University Hospital Policlinico-San Marco, University of Catania, 95124 Catania, Italy; andreavescio88@gmail.com (A.V.); danilozuccala90@gmail.com (D.Z.); vpetrantoni1@gmail.com (V.P.); amico_mirko87@hotmail.com (M.A.); giusessa@unict.it (G.S.); vitopavone@hotmail.com (V.P.); 2 Department of General Surgery and Medical Surgical Specialties, Urology Section, University Hospital Policlinico-San Marco, University of Catania, 95124 Catania, Italy; giorgioivan.russo@unict.it

**Keywords:** sarcopenia, hip fracture, diagnosis, treatment, prevention, dual-energy X-ray absorptiometry, bisphosphonate, β-hydroxy-β-methylbutyrate, exercise intervention

## Abstract

Background: Sarcopenia is defined as a progressive loss of muscle mass and muscle strength associated to increased adverse events, such as falls and hip fractures. The aim of this systematic review is to analyse diagnosis methods of sarcopenia in patients with hip fracture and evaluate prevention and treatment strategies described in literature. Methods: Three independent authors performed a systematic review of two electronic medical databases using the following inclusion criteria: Sarcopenia, hip fractures, diagnosis, treatment, and prevention with a minimum average of 6-months follow-up. Any evidence-level studies reporting clinical data and dealing with sarcopenia diagnosis, or the treatment and prevention in hip fracture-affected patients, were considered. Results: A total of 32 articles were found. After the first screening, we selected 19 articles eligible for full-text reading. Ultimately, following full-text reading, and checking of the reference list, seven articles were included. Conclusions: Sarcopenia diagnosis is challenging, as no standardized diagnostic and therapeutic protocols are present. The development of medical management programs is mandatory for good prevention. To ensure adequate resource provision, care models should be reviewed, and new welfare policies should be adopted in the future.

## 1. Introduction

Sarcopenia-related falls and fractures play an important role in our society due to the increased average age of the population [1]. Hip fractures are becoming an evolving and more current problem, as well as one of the most serious medical and social concerns. Hip fractures result in enhanced mortality, perpetual physical morbidity and reduced activities of daily living (ADL) [2,3], with a decrease of the quality of life for caregivers and an increased economic impact on society and government spending [4,5,6]. Nowadays, the prevention of hip fractures is considered crucial for preserving an acceptable quality of life in older patients. For these reasons, the role of the muscles trophism and function is crucial to prevent traumas in older patients [1]. Ageing is inversely related to the mass and strength of skeletal muscles, and their loss accelerates after 65 years of age, leading to an increased risk of adverse outcomes [7]. For the last 30 years, a considerable effort has been made to understand the condition of sarcopenia, and several definitions have been proposed. Sarcopenia was first defined by Rosenberg as an age-associated loss of skeletal muscle mass [8], but recently, it has been identified as a disease, and is included in the ICD-10 code (M62.84) [9]. Several disease descriptions were suggested during the last 20 years, but substantial operative variances are present concerning definitions, including nomenclature, the technique of assessment of lean mass, the technique of standardization of lean mass to body size, cut-points for weakness and cut-points for slowness [10]. One of the most accepted was described by the EWGSOP (European Working Group on Sarcopenia in Older Persons), updated in 2018 (EWGSOP2), considering sarcopenia as a “progressive loss of muscle mass and muscle strength, associated to an increased likelihood of adverse events, such as falls, fractures, physical disability and death” [7]. Several authors investigated the differences in sarcopenia cases, agreeing with EWGSOP1 and EWGSOP2 and noting substantial discordance and limited overlap of the definitions [11,12]. Nevertheless, the EWGSOP2 is crucial suggestion to evaluate a possible condition of sarcopenia by measuring the muscle strength, muscle mass and physical performance [13]. Aging is related to variations in body structure and uncontrolled weight loss. The progressive loss of skeletal muscle mass (SMM) and strength promotes functional and physical disability, leading to poor quality of life [7]. The body composition changes were reported in several studies [7,14,15]. Cruz-Jentoft et al. [7] showed a loss of muscle strength in older patients through measurement grip strength with a dynamometer. Hida et al. [14] demonstrated a greater sarcopenia prevalence and more diminished leg muscle mass in subjects following a hip fracture than uninjured subjects with the same age. The most efficient technique to date, dual energy X-ray absorptiometry (DXA), assesses lean mass [16]. Bioelectrical impedance analysis (BIA), CT, and MRI can be used in selected cases [16]. DXA has several advantages, including low cost, low irradiation exposure and easy availability and usability. However, the difficulty of performing this examination in patients with hip fracture or in subjects undergoing recent orthopaedic surgery, due to post-surgical pain and immobility, the use of machines with non-uniform calibrations between them and the lack of universally shared protocols, makes DXA not always reliable in the quantification of MM and in the instrumental diagnosis of sarcopenia [11,17]. No specific drugs have been approved for the treatment of sarcopenia and the literature lacks evidence [16]. Research activity is focused on developing new drugs for sarcopenia, although progress has not been straightforward. Initial interest in selective androgen receptor modulators is related to small phase I and II trials [18,19]. For these reasons, the interest in sarcopenia is rising in orthopaedic surgery, due to the high prevalence of older patients, especially those suffering for hip fractures [20], and sarcopenia could be considered as a hip fracture risk factor.

The aim of this systematic review was to analyse diagnosis methods of sarcopenia in patients with hip fracture and evaluate prevention and treatment strategies described in literature.

## 2. Experimental Section

### 2.1. Study Selection

From their date of inception to 19th March 2020, two independent authors (AV and GT) systematically reviewed the main web-based databases, Science Direct and PubMed, agreeing to the Preferred Reporting Items for systematic Reviews and Meta-Analyses (PRISMA) recommendations [19]. The research string used was “sarcopenia AND (diagnosis OR treatment OR prevention) AND (femoral neck fracture OR hip fracture)”. In order to extract the number of patients, mean age at treatment, sex, type of treatment, follow-up, and year of the study a standard data entry form was used for each included original manuscript. Three independent reviewers (MA, PV and DZ) performed the quality evaluation of the studies. Discussing conflicts about data were resolved by consultation with a senior surgeon (VP).

### 2.2. Inclusion and Exclusion Criteria

Eligible studies for the present systematic review included sarcopenia diagnosis, treatment and prevention in hip-fractured patients. The original titles and abstracts examination were selected using the following inclusion criteria: Sarcopenia, hip fractures, diagnosis and treatment and prevention with a minimum average of 6-months follow-up in last 20 years. The exclusion criteria were: Patients’ cohort with no sarcopenia diseases, less than 6 months of symptoms and no human trials. Each residual duplicate, articles related on other issues or with inadequate technical methodology and available abstract were ruled out.

### 2.3. Risk of Bias Assessment

According to the ROBINS-I tool for nonrandomized studies [21], a three-stage assessment of the studies included risk of bias assessment was performed. The first step involved the design of the systematic review, the next phase was the assessment of the ordinary bias discovered in these manuscripts and the final was about the total risk of bias. “Low risk” and “Moderate risk” studies were considered acceptable for the review. The assessment was separately performed by three authors (MA, PV and DZ). Any discrepancy was discussed with the senior investigator (VP) for the final decision. All the authors agreed on the result of every stage of the assessment.

## 3. Results

### 3.1. Included Studies

**Figure 1:**
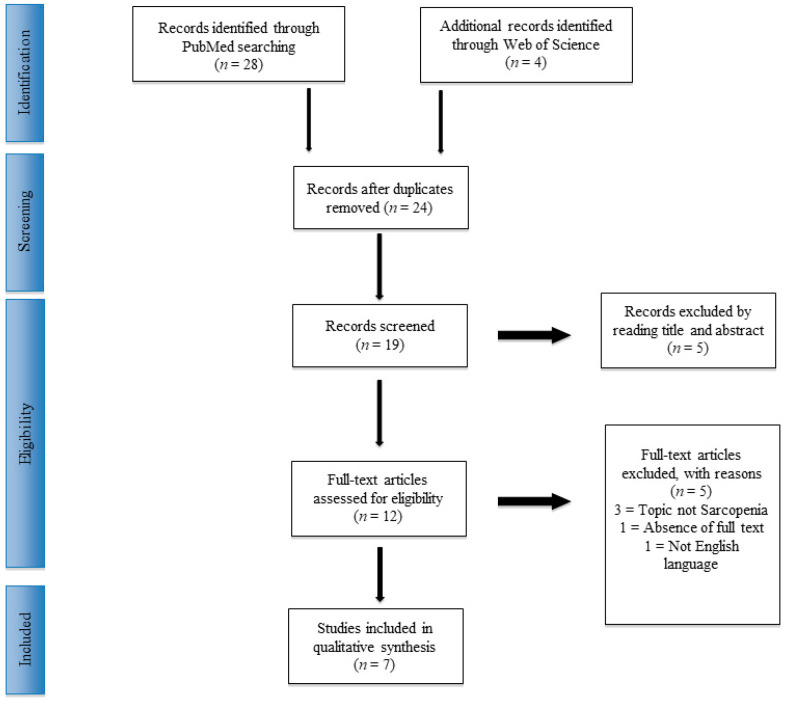
PRISMA (Preferred Reporting Items for Systematic Reviews and Meta-Analysis) flowchart.

Thirty-two manuscripts were recovered. Twenty-four articles were chosen, following the exclusion of duplicates. At the end of the first screening, according to the selection criteria previously described, nine articles were chosen as eligible for full-text reading. Metanalysis or systematic reviews were eliminated from the study. Finally, after reading the complete articles and examining the reference list, we chose seven manuscripts comprised of randomized controlled human trials (hRCT), prospective and retrospective cohort or series studies, according to previously described criteria. A selection and screening method PRISMA [22] flowchart is provided in Figure 1.

### 3.2. The Diagnosis of Sarcopenia in Patients Affected by Hip Fracture

Kramer et al. [23] performed biopsies of vastus lateralis to assess the muscle changes. The sample was divided in to three groups: Healthy young women (HYW) (18–25 years), healthy older women (HEW) (>65 years) and older women (>65 years) affected by traumatic hip fracture (FEW). FEW Type 2 fibers (2.609 ± 185 µm^2^) were noted significantly smaller compared to HEW (3.723 ± 322 µm^2^; *p* = 0.03) and HYW (4.755 ± 335 µm^2^; *p* < 0.001).

Hansen et al. [16] compared the Computed Tomography (CT) and dual-energy X-ray absorptiometry (DXA) efficiency in the assessment of midthigh muscle mass (SMM) and midthigh cross-sectional area (CSA) respectively, after a hip fracture with 12 months follow-up. The two measures were significantly linked to baseline (r = 0.86, *p* < 0.001). Ratios of midthigh fat to lean mass were comparably related (interclass correlation coefficient = 0.87, *p* < 0.001). Data of the change from baseline to follow-up showed a low correlation (interclass correlation coefficient = 0.87, *p* = 0.019). The assessment of muscle mass by DXA-derived midthigh slice has been shown to be reasonably accurate in comparison to a single-slice CT technique in this sample of frail older patients.

Villani et al. [24] evaluated the agreement degree between DXA and bioelectrical impedance spectroscopy (BIS) associated to corrected arm muscle area (CAMA). No significant changes (*p* = 0.78) were found when comparing fat-free mass (FFM) with BIS (FFMBIS) to FFM with DXA (FFMDXA) mean bias. Nevertheless, when included as an independent covariate, gender demonstrated an influence on variation in the mean bias over time (*p* = 0.007). The influence of BMI had no effect on change in the mean bias (*p* = 0.19). Similarly, no significant changes in the mean bias were observed between SMMDXA and SMMCAMA across each assessment time point (*p* = 0.18). At each assessment follow-up, both the techniques were observed overestimated SMM and FFM.

### 3.3. Treatment of Sarcopenia in Patients Affected by Hip Fracture

Flodin et al. [25] evaluated the efficacy of nutritional supplementation on body composition (BC), handgrip strength (HGS) and health-related quality of life (HRQoL) in 79 hip-fractured patients (mean age 79 ± 9 years). Patients were divided into a protein and bisphosphonate group (PB) group, bisphosphonate-only group (BO) and a control group (CG) with 12 months follow-up. All groups included the CG, received calcium and Vitamin D supplementation. No significant differences in changes of FFM Index, HGS and HRQoL were detected during the follow-up period between the groups.

Invernizzi et al. [26] assessed the essential amino acid supplementation (AAS) in hip-fractured patients. Thirty-two patients (sarcopenia-affected = 71.9%) underwent to a 2-month rehabilitative protocol combined with dietetic counselling. The AA group (16 subjects) had an AAS, while the NAA group did not receive AAS. According to Janssen criteria, both groups were divided in subgroups: Sarcopenic (Sac) and non-sarcopenic (No-Sac) patients. At 2 months follow-up, the Sac AA group (*n* = 10) obtained better significant results in the Iowa Level of Assistance scale (ILOA) and all the primary outcomes (*p* < 0.017) compared to Sac NAA cohort (*n* = 13). The No-Sac groups had similar results.

Malafarina et al. [27] investigated the effectiveness of β-hydroxy-β-methylbutyrate (HMB) oral NS on muscle mass and nutritional markers (BMI, proteins) in patients >65 years with hip fracture. Fifty-five patients (IG) received standard diet plus HMB NS and 52 patients (CG) received standard diet only. The authors used the EWGSOP criteria to diagnose sarcopenia and its prevalence among the entire population was 72%. The sarcopenia diagnostic markers were gait speed (GS), HGS and BC (assessed with BIA). Positive results were recorded in IG for grip work index (*p* = 0.188), muscle mass (MM) (*p* = 0.031) and appendicular lean mass (aLM) (*p* = 0.020). GS analysis did not show a significant difference (*p* = 0.367).

### 3.4. Prevention of Sarcopenia in Patients Affected by Hip Fracture

In a study by Ding-Cheng Chan et al. [28], 110 patients over 50 years of age with high-risk fracture underwent 3-month exercise interventions. According to different modalities of the exercise, the cohort were randomly divided into integrated care (IC) group and machine-based low extremities exercise (LEE) group. The authors observed a gain in limb mass in the entire cohort (1.13%, *p* < 0.05) with a significant change in the LEE group (1.13%, *p* < 0.01). Both groups obtained significant improvement in muscle strength measured with curl, press and leg extension, grip strength, gait speed, chair stand test and time up and go test. Improvements were seen in leg curl in the LEE group (29.78%, *p* = 0.001).

The most important results of the included articles were summarized (Table 1).

## 4. Discussion

### 4.1. General Considerations and Key Findings

According to the review findings the diagnosis is still a challenge. The lack of an optimal instrumental tool for diagnosis in hip-fractured patients demonstrates the crucial role of physicians in these cases. The diagnosis is not instrumental data but the correct analysis of the clinical examination and patients’ physical status evaluation in association with the results of the tool. At the same time, the nutritional supplementation and hip fracture prevention exercise program are mandatory to avoid the variances in body composition after midlife. Therefore, body composition evaluation is a crucial element for measuring health status in older adults.

The higher incidence of fractures, especially in the spinal column and femoral neck, is attributable to the condition of osteopenia or osteoporosis. Several authors have debated the correlation of bone mineral density (BMD) to muscle mass (MM). However, this association is controversial. Gillette-Guyonnet et al. [29] and Walsh et al. [30] claimed there was no muscle–bone relationship. On the other hand, Locquet et al. exhaustively explored the correlation between muscle and identified a subpopulation affected by the reduction in bone and muscle mass [31]. Moreover, Hirschfeld et al. suggested considering the two condition as a new pathologic disorder, where the subjects affected should be defined as “osteosarcopenic patients” [32]. The controversial findings should be explained by the different diagnosis protocols used. In fact, the sarcopenia diagnosis is often challenging, and there is not an instrumental method or standard algorithm commonly accepted for the evaluation. EWGSOP2 suggests combining clinical tests and instrumental investigations to evaluate the muscle strength, physical performance and muscle mass [11].

### 4.2. Sarcopenia Diagnosis in Hip-Fractured Patients

Determining grip strength is easy, inexpensive and routine in clinical practice. The evaluation requires calibrated handheld dynamometer use under well-definite exam circumstances with interpretive data from appropriate reference populations [11,33]. On the other hand, the technique measurements can be influenced by the examiner [33]. Similarly, the chair stand test (also called the chair rise test), aims to assess the quantity of time that the patient needs to rise five times from a seated position without using their arms [30]. The Gait Speed test is helpful in the evaluation of physical performance. The principles are the Short Physical Performance Battery (SPPB), and the Timed-Up and Go test (TUG), but the results can be influenced by patient compliance. The Gait Speed test is a rapid, secure and reliable test to assess sarcopenia by EWGSOP2 [11]. The patient walks for 4 m while the clinical staff records the walking speed using an electronic device or manually with a stopwatch. A Gait Speed of ≤0.8 m/s is a severe sarcopenia marker [34,35,36]. The SPPB is a complex test aimed to analyse gait speed using a balance test and a chair stand test. The highest score is 12 points, and a score of ≤8 points suggests inadequate physical performance. The TUG test assesses the taken time to rise from a standard chair, walk 3 m away, turn around, walk back and sit down again. A score of >20 s is indicative of poor physical performance [37]. 

Due to the reduced mobility in the hip-fractured patients, and consecutively, to the impossibility in performing the main tests used to assess the disease, the instrumental tools are important part of diagnosis, even if they can replace the clinical evaluation. 

DXA is a more widely accessible tool to establish MM [38], and can be defined as total body SMM, as ASM or as muscle cross-sectional area of specific muscle groups [16]. New methods have been studied, including midthigh muscle measurement by CT or MRI, BIS, psoas muscle measurement with CT, the detection of specific biomarkers and other tests [16,24,25]. CT and MRI allow for a precise and detailed study of soft tissues and they offer reliable and universally shared data. On the other hand, these methods have a high cost and it is difficult to find institutes where it is possible to quickly perform them. Moreover, CT exposes patients to a high rate of irradiation [16,34]. Hansen et al. [16] compared SMM estimated by DXA to midthigh muscle CSA, determined by CT, in a group of older patients with hip fracture, observing a positive correlation between CT-determined midthigh muscle CSA and DXA-derived midthigh SMM. The assessment of MM and body composition by DXA-derived midthigh slice has been shown to be reasonably accurate in comparison to a single-slice CT technique in this sample of frail older patients [16].

BIS is another technique used to estimate SMM. The measurement is not a direct evaluation of MM, but an estimation on the whole-body electrical conductivity, through conversion equations [37]. BIS needs highly trained personnel, and the institutes where it can be performed are very difficult to find. Villani et al. [24] compared BIS associated to CAMA and DXA, noting BIS were reliable, but the difficulties in carrying out the examination and in the use of conversion equations led to DXA as the preferred reference technique. Muscle mass evaluation is not the only parameter that can be associated to sarcopenia. A low muscle quality is considered as one of the diagnosis criteria by EWGSOP [11]. Muscle quality is one of the main determinants of muscle function, depending on different factors (fibre composition, architecture, metabolism, intermuscular adipose tissue, fibrosis, motor unit activation) [39]. In particular, the decline of type II muscle fibres (II-MF) is responsible for muscle mass reduction [40].

Kramer et al. [22] performed vastus lateralis biopsies in different groups, confirming a significant II-MF atrophy in older women with hip fractures when compared to healthy older or young women. Since muscle atrophy is associated to loss of function, the author suggested that II-MF atrophy could lead to a higher risk of falls and consequent fractures. This study has some limits. There was no measure of strength and the sample was exclusively female, but the findings could be relevant to treat sarcopenia and to understand the II-MF atrophy causes. The histological diagnosis of sarcopenia could be a valuable way to understand physiopathology of sarcopenia in patients with hip fractures, even if it is not obviously suitable for routine diagnosis.

### 4.3. Sarcopenia Treatment in Hip-Fractured Patients

The treatment of sarcopenia in patients affected by hip fractures is a multidisciplinary challenge and, according to our findings, great attention should be given to nutritional status. Malnutrition is a highly prevalent condition in the geriatric population affected by this fracture [27]. Therefore, oral nutritional supplementation (ONS), in addition to rehabilitation programs, has become the subject of debate between different authors. Flodin et al. [25] investigated the effects of protein-rich supplementation and bisphosphonate on body composition, handgrip strength and quality of life in patients with hip fracture at 12-months follow-up. In a group, the combination of bisphosphonates and protein supplementation had no significant effects on handgrip strength (HGS), body composition and health-related quality of life (HRQoL). In another group, a positive effect of protein-rich supplementation and bisphosphonates on HGS and HRQoL was demonstrated.

Malafarina et al. [27] showed good results using oral nutritional supplementation with β-hydroxy-β-methylbutyrate (HMB). This approach improves MM, function and general nutritional status in hip-fractured patients [27]. HMB, a metabolite of leucine, has beneficial effect on MM and function in older people [41], but considering the lack of evidence focused on hip-fractured people, more investigations are needed in the treatment of sarcopenia with HMB in these patients. On the other hand, the role of a nutritional intervention without exercise for the treatment of sarcopenia is debated [41]. Although many studies have described good results in increasing protein intake in the older population [42,43], the timing and distribution is unclear [44]. 

### 4.4. Sarcopenia Prevention in Hip-Fractured Patients

Despite the few studies focused on sarcopenia prevention in our study, it could be considered the major area of research for future clinical activity and observational epidemiological trials [39] in order to identify and modify the sarcopenia risk factors. A midlife lifestyle approach could be more proper to limit the sarcopenia incidence [45].

Physical activity programs have been suggested as a relevant technique in reducing the risk of hip fracture in older patients [46,47]. In the study by Piastra et al. [47], data showed a significant improvement in MM, muscle mass index, and handgrip strength in muscle reinforcement training group, demonstrating that a muscle reinforcement program moved participants from a condition of moderate sarcopenia at baseline to a condition of normality. Ding-Cheng Chan et al. [28] evaluated effects of programs in community-dwelling older adults with high risk of fractures (> or =3% for hip fracture). The exercise authors clarified the lack of differences in the types of exercise to improve sarcopenia when compared an integrated care model to a lower extremity exercise model. However, several authors promoted rehabilitation protocols for hip-fractured patients, consisting of oral nutritional supplementation with proteins and amino acids and exercise programs [46,47]. Singh et al. [47] proposed a new rehabilitation protocol in the older with hip fracture after orthopaedic surgery. The 12-month rehabilitation program was characterized by a high-intensity progressive resistance training and a targeted treatment of balance, osteoporosis, nutrition, vitamin D and calcium, depression, home safety and social support. The authors showed a statistically significant reduction in mortality, nursing home hospitalization and disability, especially in those subjects with a systematic good health status.

A life course approach to prevention is paramount and offers chance to intervention when lifestyle changes, inspiring the increase of physical activity with immediate to lifelong advantages for skeletal muscle health [16]. 

### 4.5. Limits of the Study

The limits of the study are represented by the heterogenicity of the definition of sarcopenia and by the tools considered to assess the patient functional outcome. We extensively searched and identified all relevant last 20 years sarcopenia diagnosis-, treatment- and prevention-related articles. Therefore, risk of bias assessment showed moderate overall risk, which could influence our analysis. Moreover, in the diagnosis section, only instrumental tool evaluations without clinical assessment were detected.

## 5. Conclusions

Sarcopenia is a physiological condition and contributes to the increased risk of falls and hip fractures in the older population. However, the diagnosis of sarcopenia is challenging, especially in hip-fractured patients, and there are currently no standardised diagnostic and therapeutic protocols. The development of medical management programs is mandatory for good prevention. To ensure adequate resource provision, care models should be reviewed, and new welfare policies should be adopted in the future. 

## Figures and Tables

**Table 1:** Included studies summary. Dual-energy X-ray absorptiometry (DEXA); healthy young women (HYW); healthy elderly women (HEW); elderly women with a hip fracture (FEW); Dual-energy X-ray absorptiometry (DEXA); BIS (bioletrical impedance spectroscopy); corrected arm muscle area (CAMA); Fat-free mass (FFM) with BIS (FFMBIS); FFM with DXA (FFMDXA); handgrip strength (HGS) and health-related quality of life (HRQoL); Timed Up and Go test (TUG); Iowa Level of Assistance scale (ILOA); Mini Nutritional Assessment−Short Form (MNA-SF); Barthel index (BI); Functional Ambulation Categories (FAC).

Author	Sample	Intervention	Outcome Measures	Results	Limits of the Study
Kramer et al., 2017	15 HYW (age: 20.3 ± 0.4 years), 15 HEW (age: 78.8 ± 1.7 years), and 15 FEW (age: 82.3 ± 1.5 years)	Muscle biopsies and immunohistochemistry	Muscle fibre type distribution, myonuclear and satellite cell content	FEW resulted in atrophy of muscle fibres Type I and II, associated to a general deterioration in muscle fibres Type II size. Atrophy of Type II muscle fibre in these subjects is associated to a decrease in myonuclear content of Type II muscle fibre.	No measures of muscle mass and/or strength. No data about men
Hansen et al., 2007	30 patients over 60 years old with hip fractures affected in community living patients (not nursing houses, no dementia, no terminally ill)	DEXA-derived midthigh slice has been found to be reasonably accurate in comparison with a single-slice CT technique	Muscle mass and composition	Superior accessibility and simplicity of DEXA utilize. DEXA errors inherent suggest that it should be used to studying groups of patients rather than individuals and in longitudinal trials.	Patients with non-traumatic neck hip fracture
Villani et al., 2012	79 Patients with hip fracture, free in the community.	BIS; DEXA	FFM and SMM, and CAMA	BIS demonstrated sufficient agreement against DXA.	Predictive power and Repeatability
Flodin et al., 2015	79 patients divided in 3 groups: Group N (26 patients); Group B (28 patients); Group C (25 patients)	Group N: 40 g of protein and 600 kcal combined with risedronate and calcium 1 g and vit D 800 IE; Group B: Same of Group N + bisphosphonates alone once weekly for 12 months; Group C: Control. All groups received conventional rehabilitation	Body composition; HGS; HRQoL at 0, 6 and 12 months postoperatively	No considerable variation in baseline attributes was observed between the groups. There was a positive correlation between FFMI and aLMI, r = 0.92, *p* < 0.01.	Small number of study subjects. The use of different devices of DXA measurements inflict uncertainties on the validity of the results
Invernizzi et al., 2018	32 patients over 65 years old divided in two groups: Sarcopenic and non-sarcopenic.	Physical exercise rehabilitative programme and received a dietetic counselling. One group was supplemented with two sachets of 4 g/day of essential amino acids.	HGS, TUG, ILOA, Nutritional assessment, HRQoL baseline (T0) and after 2 months of treatment (T1)	At T1 follow-up, statistically significant differences in all the outcomes (*p* < 0.017) in sarcopenic patient who received AA supplementation.	Small size, the use of BIA to calculate the SMI.
Malafarina et al., 2017	107 patients: Group control (CG), Group intervention (IG)	CG: standard diet (1500 kcal); IG: CG+oral nutritional supplementation (CaHMB 0.7 g/100 mL, 25(OH)D 227 IU/100 mL and 227 mg/100 mL of calcium). All patients received Physical therapy	Body composition, HGS, MNA-SF, BI, FAC	BMI and lean mass were constant in IG patients, while reduced in the CG. The vitamin D and proteins and concentration had improved more in the IG than in the CG. ADL recovery of was more frequent in the IG (68%) than in the CG (59%) (*p* = 0.261)	Patients received physiotherapy 5 days por week. No follow-up after discharge. Diagnostic criteria for sarcopenia.
Chan et al., 2018	110 participants divided in Integrated group (IG) and Low extremity group (LEG)	IC: 15 min warm-up + Resistance training (30 min) + Balance training (10 min) at least once a week for 12 weeks. LEG: 12-week machine based lower extremity resistance exercise twice per week (30 min each). All participants had received a lecture on prevention of osteoporosis, sarcopenia and fall-related injury	Body composition; Gait speed (m/s), chair stand test and timed up-and-go test. Hip and L-spine BMD.	Decrease in weight (*p* < 0.01) and limb fat (*p* < 0.001) were noted in IC group. Im LLE group, Significant variations were detected in limb mass (*p* < 0.01). No variation in the cohorts regarding change on body composition. Significant enhancement in muscle strength in both the cohorts. After 3 months, significant improvement for leg strength but higher gain in LEE on leg curl performance (*p* = 0.001). BMD of L-spine improved but similar after 3 months.	BMD tests were not strictly performed on all participants.
